# Soluble RAGE but not endogenous secretory RAGE is associated with albuminuria in patients with type 2 diabetes

**DOI:** 10.1186/1475-2840-6-9

**Published:** 2007-03-07

**Authors:** Per M Humpert, Zdenka Djuric, Stefan Kopf, Gottfried Rudofsky, Michael Morcos, Peter P Nawroth, Angelika Bierhaus

**Affiliations:** 1 Department of Medicine I and Clinical Chemistry, University of Heidelberg, Germany

## Abstract

**Background:**

Total circulating soluble receptor for advanced glycation endproducts (sRAGE) and a more defined endogenous secretory splice variant of the receptor (esRAGE) were shown to be associated with different markers of cardiovascular risk in patients with diabetes. Since previous data were partly divergent, the aim of this study was to compare sRAGE and esRAGE in a head-to-head analysis in patients with type 2 diabetes (T2DM) with albuminuria.

**Methods:**

sRAGE and esRAGE were studied in plasma of 110 T2DM patients using enzyme-linked immunosorbant assays (ELISA) detecting either sRAGE or esRAGE only. Both sRAGE and esRAGE were compared with regard to applicability as markers for vascular disease and glucose control in T2DM.

**Results:**

In bivariate analysis, sRAGE correlated with age (R = 0.22, p = 0.02) and the 24 hour albumin excretion rate (R = 0.18, p = 0.05), while esRAGE correlated positively with age only (R = 0.23, p = 0.02). In contrast to previous reports, neither sRAGE nor esRAGE correlated with glucose control or intima-media-thickness (IMT) as a predictor of macrovascular disease. In multivariate regression models, the associations between sRAGE and albuminuria as well as esRAGE and age were shown to be independent of glucose control, diabetes duration, body-mass index, glomerular filtration rate, blood pressure and gender.

**Conclusion:**

This is the first study comparing sRAGE and esRAGE as markers of vascular complications in patients with T2DM. sRAGE but not esRAGE is independently associated with albuminuria in these patients while neither sRAGE nor esRAGE are associated with markers of glucose control or macrovascular disease.

## Background

Soluble forms of the receptor for advanced glycation endproducts (sRAGE) were previously shown to appear in human blood and to be associated with glucose control as well as vascular risk factors in diabetes mellitus and the metabolic syndrome [1-6]. Plasma sRAGE consists of an endogenous splice variant of RAGE lacking the transmembrane domain of the receptor (esRAGE) [7] as well as proteolytically cleaved forms shed into the bloodstream by action of extracellular metalloproteinases [8,9]. Both sRAGE and esRAGE were shown to act as decoys binding inflammatory RAGE ligands like advanced glycation endproducts (AGEs) that accumulate in diabetes mellitus [1,7,9,10]. It is speculated, that the soluble forms of RAGE might counteract inflammatory reflexes triggered by RAGE ligands such as AGEs, S100 proteins and HMGB1 [1]. However, it seems questionable that the circulating forms of RAGE exert a biological effect, since the sRAGE concentrations found in plasma are ~ 1000 times lower than needed for the binding of AGEs [10]. Nevertheless, associations of sRAGE and esRAGE with different aspects of metabolic, vascular and autoimmune disease might make them valuable risk markers [1-6,11,12].

Two ELISA assays for the detection of circulating RAGE are commercially available using antibodies that detect total circulating sRAGE or recognize esRAGE only [13]. The previously published studies using these assays reported inconsistent data with respect to the association of sRAGE and esRAGE with diabetes and glucose control. While one study detected increased levels of sRAGE [3], another study described decreased levels of esRAGE in patients with type 1 diabetes [5]. Likewise, esRAGE was associated with markers of glucose control in one study [6] while a correlation of sRAGE with measures of glucose control was missing in another study of patients with type 2 diabetes [4]. These reports focussed on associations of either esRAGE or total sRAGE with disease markers, yet, it seems likely that sRAGE and esRAGE are distinct markers since the secretion of esRAGE is a consequence of RAGE mRNA processing [7]. In contrast, sRAGE is a sum of esRAGE and RAGE most likely shed upon digestion by metalloproteinase action on the cellular surface [1,8]. Hence, we conducted a head-to-head analysis in patients with T2DM and albuminuria to compare associations of both total sRAGE and esRAGE with markers of glucose control and vascular risk.

## Methods

The study was approved by the local ethics committee; 110 T2DM patients were recruited from family practices being referred to our diabetes outpatient clinic for specialist treatment after giving written consent. For eligibility, patients had to be tested positive for albuminuria in two separate spontaneous urine samples (> 20 mg/dl albumin). Patient characteristics are given in Table 1. 24-hour urine samples were collected on three consecutive days and the mean of albumin excretion was taken for statistical evaluation. All blood values as well as ambulatory 24-hour blood pressure values (given as mean of 24 hours) were taken on the day of study entrance. Glomerular filtration rate (GFR) was estimated using the Cockroft-Gault formula [14]. IMT was detected non-invasively in the enddiastolic phase of the heart cycle in the far wall of the common carotid artery approximately 2 cm distal of the carotid bulb on both sides using high resolution ultrasound. 4 measurements were taken on each side and the mean was calculated for statistical analysis. Total sRAGE antigen (R&D Systems, Wiesbaden, Germany) and esRAGE (B-Bridge International, Sunnyvale, USA) antigen were detected in plasma in duplicates by Elisa according to the manufacturer's instructions. For statistical evaluation, variables were correlated using Spearman's Coefficient. A stepwise multivariate linear regression model was calculated to detect independent associations of age, gender, diabetes duration, BMI, HbA1c, fasting glucose, GFR, mean 24 h blood pressure and 24 hour albumin excretion with sRAGE and esRAGE levels. p ≥ 0.1 for F-values was taken as criterion for exclusion of variables. Albumin excretion, sRAGE and esRAGE variables were not normally distributed and log-transformed for multivariate analysis. SPSS 11.0 software was used for all statistical testing (SPSS, Chicago, Illinois, USA).

**Table 1:** Patient characteristics and associations of sRAGE and esRAGE with different variables in 110 patients with type 2 diabetes

		**sRAGE**^#^		**esRAGE**^#^	
	Characteristics	univariate (R)	multivariate (β)	univariate (R)	multivariate (β)

Age (years)	59 ± 7	0.22*	**-**	0.23*	0.24**
Gender (f/m)	24/86	-	0.21*	-	**-**
Diabetes duration (years)	13 ± 8	0.11	-	0.11	-
BMI (kg/m^2^)	33 ± 6	0.04	-	-0.04	-
HbA1c (%)	7.4 ± 1.2	-0.02	-	0.05	-
Fasting glucose (mmol/l)	8.2 ± 3.0	0.05	-	0.05	-
Glomerular filtration rate (ml/min)	128 ± 50	-0.15	-	-0.14	-
mean 24 h systolic BP (mmHg)	141 ± 16	0.02	-	-0.06	-
mean 24 h diastolic BP (mmHg)	80 ± 8	-0.14	-	-1.8	-
24 h Albumin excretion (mg)^#^	170 ± 406	0.18*	0.22*	0.10	**-**
Macrovascular Complications (%)	33				
Retinopathy (%)	27				
Polyneuropathy (%)	52				
Insulin therapy (%)	64				
ACE-Inhibitors/AT-R antagonists (%)	88				

Data is given as mean ± SD. R = Spearman correlation coefficient. β = standardized coefficient as given by stepwise multivariate regression analysis. # = variables were not normally distributed and log-transformed for multivariate analysis. * p ≤ 0.05, ** p ≤ 0.01

## Results

**Figure 1:**
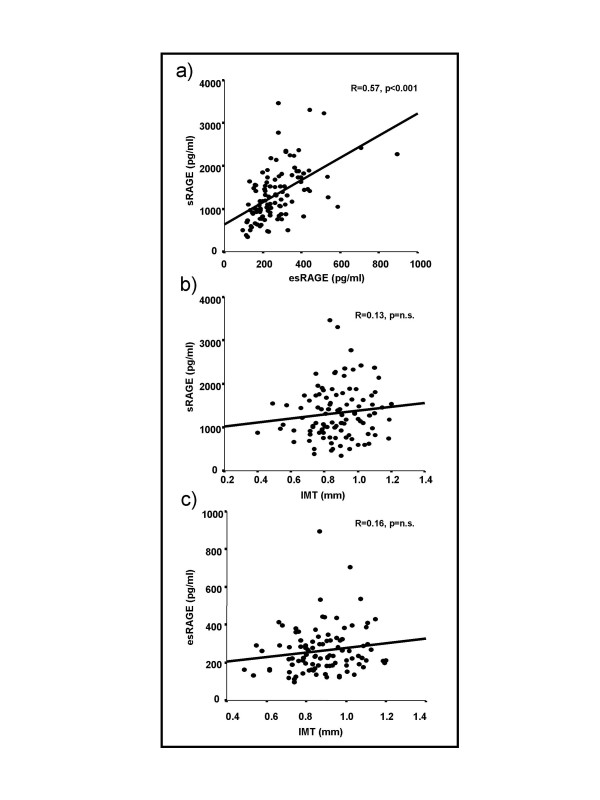
Bivariate correlations of sRAGE and esRAGE (a), sRAGE and IMT (b) as well as esRAGE and IMT (c) in patients with T2DM. R = Spearman's correlation coefficient. IMT = intima-media thickness as detected by high resolution ultrasound.

sRAGE and esRAGE concentrations were detected at 1326 ± 621 pg/ml and 269 ± 26 pg/ml respectively; sRAGE and esRAGE were significantly and positively correlated (R = 0.57, p < 0.001; Fig. 1a). Multivariate adjustment in a stepwise regression model including esRAGE, age, gender, body-mass-index, albumin excretion rate and HbA1c showed an independent association for esRAGE with sRAGE only. The model applied explained ~ 34% of the variation in total sRAGE (β = 0.58, p < 0.001, R^2 ^= 0.34; not shown). In bivariate analysis, sRAGE correlated with age (R = 0.22, p = 0.02) and the 24 hour albumin excretion rate (R = 0.18, p = 0.05; Table 1) while esRAGE correlated positively with age only (R = 0.23, p = 0.02; Table 1). In multivariate regression models, the associations between sRAGE and 24 hour albumin excretion (β = 0.22, p = 0.02; Table 1) as well as esRAGE and age (β = 0.24, p = 0.01, Table 1) were independent of glucose control, diabetes duration, body-mass-index, glomerular filtration rate, blood pressure and gender. However, in contrast to previous reports [2,3,5], in our cohort neither sRAGE nor esRAGE correlated significantly with glucose control (Table 1) or IMT as a predictor of macrovascular disease (Figure 1b,c). Noteworthy, neither subtraction of esRAGE from sRAGE nor the esRAGE/sRAGE ratio resulted in significant associations with albuminuria, IMT or markers of glucose control (not shown). In addition, sRAGE and esRAGE did not correlate with the glomerular filtration rate and the latter did not have any influence on associations of sRAGE and esRAGE with markers of disease studied in the multivariate model (Table 1). Patients treated with angiotensin converting enzyme (ACE) – inhibitors or angiotensin receptor (AT-R) – antagonists had significantly higher sRAGE and esRAGE levels (1370 vs. 997 pg/ml, p < 0.01 and 278 vs. 198 pg/ml, p < 0.01) [15]. Hence, additional multivariate models including sRAGE/esRAGE as dependent variables and age, 24 hour albumin excretion, glomerular filtration as well as ACE – inhibitor/AT-R antagonist treatment were performed. In these models, sRAGE showed marginally significant independent associations with age (β = 0.23, p = 0.05) and 24 hour albumin excretion (β = 0.18, p = 0.05) while esRAGE was independently associated with age only (β = 0.23, p = 0.05).

## Conclusion

In this first comparative study of sRAGE and esRAGE as markers of disease in type 2 diabetes, esRAGE concentrations were found to be ~ 5 times lower than total sRAGE concentrations, but correlated significantly with sRAGE. In the multivariate regression model mentioned above, esRAGE explained one third of the variation in sRAGE found in our cohort suggesting diverse mechanisms leading to appearence of sRAGE and esRAGE in the circulation. sRAGE is most likely cleaved from the cell surface by action of matrix metalloproteinase 9 [1,8], which is known to be upregulated and precedes the development of albuminuria in patients with T2DM [16]. Consistently and in line with previous data [4,17], we found a weak but independent association of sRAGE with albuminuria in the multivariate model with gender being the only additional independent variable influencing sRAGE levels. There were no associations of esRAGE with the albumin excretion rate. In view of the findings that AGEs induce the expression of RAGE [18] and that serum AGEs correlate positively with AGEs in T2DM [17], an increase in AGEs might also cause an upregulation of esRAGE. Hence, our finding of an independent positive association of esRAGE with age (Table 1) could be consequence of an increased AGE-load during aging [19-21]. Interestingly, soluble RAGE itself might be capable of triggering inflammatory reactions via binding of Mac1 and subsequent activation of NF-κB and thus contribute to the development of vascular complications [22].

The divergent data concerning associations of sRAGE and esRAGE with markers of glucose control and vascular risk in our cohort and published cross-sectional studies are likely to be a consequence of the study designs applied. Inverse associations of sRAGE with IMT and coronary artery disease were previously described in non-diabetic and diabetic cohorts [6,23] and are not evident in our T2DM patients. The inverse correlation of esRAGE or sRAGE with the HbA1c reported in patients with type 1 diabetes [5,6] and T2DM [2] as well as the weak associations of esRAGE with IMT [6] might partly be a consequence of analyses in mixed cohorts of healthy volunteers and patients with diabetes. This leads to a bias when diabetes is not entered as an additional variable in multivariate models and is especially true, when significant differences in esRAGE levels are evident between diabetes patients and healthy controls [6]. Consistently, these data cannot be reproduced in this study and previous publications [4,17] analysing patients with T2DM separately. As previously shown in patients with type 1 diabetes [15], sRAGE and esRAGE levels were increased in our type 2 diabetes patients. This might either be a consequence of direct effects of ACE – inhibitors or AT-R antagonists on sRAGE secretion (which was shown in bovine endothelial cells in vitro [15]) or a RAGE-ligand dependent induction of esRAGE.

It cannot be excluded that significant correlations of sRAGE or esRAGE with IMT or glucose control can be found in larger groups, a valuable biomarker would however be expected to show associations even in smaller cohorts like the T2DM patients presented herein. The results argue for a distinct role of sRAGE and esRAGE as potential markers in diabetes: while total sRAGE indicates microvascular damage, plasma esRAGE is not associated with any markers of disease in T2DM. Prospective clinical trials will have to define the impact of sRAGE as a marker of cardiovascular risk in diabetes mellitus.

## Competing interests

The author(s) declare that they have no competing interests.

## Authors' contributions

PMH conceived of the study, recruited participants, analysed data and drafted the manuscript. ZD carried out the immunoassays for sRAGE and prepared patient material for analysis. SK performed immunoassays for esRAGE. GR participated in recruitment of patients and reviewed statistics. MM and PPN participated in the design of the study and reviewed the manuscript. AB conceived of the study, participated in its design and coordination and helped to draft the manuscript. All authors read and approved the final manuscript.
